# Low Concentration of Anti-Auxin and Anti-Fungal Agent Accelerates the PLB Regeneration of *Dendrobium okinawense* under Green LED

**DOI:** 10.3390/plants11081082

**Published:** 2022-04-15

**Authors:** Hasan Mehbub, Kazuhiko Shimasaki, Hasan Mehraj

**Affiliations:** 1 The United Graduate School of Agricultural Sciences, Ehime University, Matsuyama 790-8556, Japan; sujanhasan@gmail.com (H.M.); shim@kochi-u.ac.jp (K.S.); 2 Faculty of Agriculture, Kochi University, Kochi 783-8502, Japan; 3 Graduate School of Agricultural Science, Kobe University, Kobe 657-8501, Japan

**Keywords:** orchid, protocorm-like bodies, *p*-Chlorophenoxyisobutyric acid, 3-Hydroxy-5-methyl isoxazole, light-emitting diodes

## Abstract

*Dendrobium okinawense* is an endangered epiphytic orchid, and there has been no scientific report so far on its propagation. Protocorm is a mass of cells, and protocorm-like bodies (PLBs) are lookalike protocorms produced by vegetative explants in vitro. Regeneration of PLBs is a widely used technique for orchid micropropagation. We used different light-emitting diodes (LEDs) for the PLB regeneration of *D. okinawense*. The number of PLBs and fresh weight were increased by 81.1% and 80.8%, respectively, under green LED over the white fluorescent (WF) light. We added different concentrations of PCIB (p-Chlorophenoxyisobutyric acid, an anti-auxin) and HMI (3-Hydroxy-5-methyl isoxazole, an anti-fungal agent) in culture media. The number of PLBs was increased in media having 0.01 mg/L of PCIB (35.9%) compared to control (no PCIB), whereas 19.3% increased in media having 0.01 mL/L of HMI compared to control (no HMI). Green LED in combination with 0.01 mg/L of PCIB significantly increased the number of PLBs (69.0%) compared to the WF–without PCIB combination. In LEDs-PCIB and LED-HMI combinations, HMI did not show better PLBs regeneration compared with PCIB. The results suggested that a combination of low concentrations of PCIB and green LED have the potential to accelerate PLB regeneration of *D. okinawense*.

## 1. Introduction

Orchids are one of the most economically important cut- and pot-flowering plants. The seeds of the orchid are tiny, and they lack functional endosperm [1,2]. Therefore, orchids require symbiotic fungi for seed germination. Generally, very few percentages of orchid seeds (<5%) are germinated with the assistance of symbiotic fungi [1]. It requires a long period (>10 years) for the plant establishment and flowering through seed germination. Vegetative propagation of orchids is possible; however, it is a slow process and cannot generate a large number of plantlets. In vitro micropropagation is very common in orchid propagation because of the complexity in vegetative and sexual propagations. Several in vitro techniques are followed for orchid micropropagation, and propagation by PLB (protocorm-like-body) is one of them [3,4,5]. In vitro regeneration of PLB was studied in different *Dendrobium* sp. [6,7,8,9,10]. *D. okinawense* is a Japanese native epiphytic orchid closely related to *D. moniliforme* [11]. It was first identified by Hatsushima and Ida (1970) [12] from Okinawa Island in Japan.

Plant tissue culture media can be manipulated by phytoregulators such as auxin, cytokinin, and other classes to influence in vitro growth and development. PCIB (*p*-Chlorophenoxyisobutyric acid), an anti-auxin, inhibits the action of auxin that inhibits auxin- induced plant growth [13,14,15,16,17]. Polar auxin transport inhibitors can influence protocorm morphogenesis during its development [18]. HMI (3-Hydroxy-5-methyl isoxazole) or Hymexazol (Tachigaren^TM^), a member of isoxazoles, is an antifungal agrochemical. It was first launched in the 1960s to control seedling diseases and improve plant vigor in rice [19]; therefore, it is also considered a plant growth-regulating agent [20]. The multiple abilities of HMI [21] may promote the growth and development of the different plant species in vitro cultures.

Light is another key factor for in vitro propagation, and light-emitting diodes (LEDs) provide much narrower wavelengths by offering species-specific light intensity and wavelengths than conventional fluorescent light [22,23,24]. A more precise spectral quality can be selected and adjusted to the requirements of a specific plant [25]. LEDs have an enormous capacity for energy savings. Recently, LEDs have been used widely for commercial plant production and plant research [26]. The absorption of the light spectrum is species specific and depends on some other factors. A monochromatic light can function differently in dissimilar species for in vitro plant growth and development [27,28]. Some studies reported the effects of white, green, far-red, yellow, and orange LEDs on plant micropropagation [28,29,30,31,32,33], whereas LEDs showed more efficiency over fluorescent light for orchid PLB regeneration [8,9,34,35,36,37,38,39,40,41,42,43].

In Japan, *D. okinawense* has been added to the list of endangered species [44,45,46] and the legal local markets propagated it on a minor scale [47]. This small scale propagation is not sufficient for its ex-situ conservation. Availability of the micropropagation technique will make genetic conservation easier for *D. okinawense*, and it will add one more orchid species to the commercial flower market. We are reporting first time in vitro micropropagation of *D. okinawense* by PLBs regeneration. We conceptualized that the supplementation of low concentration of PCIB or HMI in culture media and then culture under specific LEDs may improve the PLBs regeneration of *D. okinawense*. Our study aimed to identify a suitable PCIB and HMI concentrations in culture media and a suitable monochromatic light to increase the in vitro PLB proliferation of *D. okinawense*.

## 2. Results

### 2.1. Evaluation of the Sucrose Concentrations

Sucrose requirement for the in vitro PLB regeneration is species specific. We evaluated the different concentrations of sucrose in the culture media (see the Section 4.4 for the methodology). We did not find any significant difference between 5, 10, 20, and 30 g/L of sucrose in culture media for the number of PLBs (Supplementary Table S1). Fresh weight of PLBs was also statistically identical between 10 and 20 g/L of sucrose (Supplementary Table S1). However, we found more numbers of shoots from 10 g/L of sucrose concentrations (Supplementary Table S1). We previously observed that 20 g/L sucrose in culture media was efficient for PLBs regeneration on *Phalaenopsis* and *Cymbidium* orchids for the 42–60 days culture periods [34,35,36,37,38,39,40,41]; therefore, we assumed the use of 20 g/L sucrose as a carbohydrate source throughout the experiments.

### 2.2. LEDs Lights for PLB Regeneration

**Figure 1:**
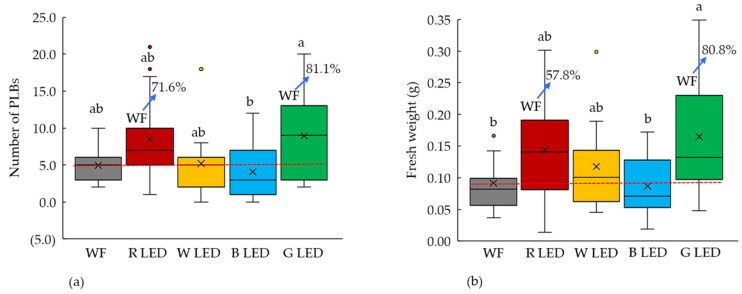
In vitro PLB regeneration of *D. okinawense* under different LEDs on (**a**) the number of PLBs and (**b**) fresh weight. WF; White fluorescent light, R; red, W; White, B; Blue, and G; Green. Crosses in the box represent the mean number of PLBs and fresh weight (total explants, *n* = 15). Red horizontal lines represent the mean mean value of control under white fluorescent light (WF). Blue arrows over the boxplot represent the percentage of increase from red horizontal line. Boxplots that share a letter are statistically identical in Tukey’s multiple comparison test (*p* < 0.05).

We evaluated the effect of four (red, white, blue, and green) LEDs on the PLB regeneration of *D. okinawense*. We found the highest number of PLBs (8.93/explants) and maximum average fresh weight (164.7 mg) from the PLBs was cultured under green LED (Figure 1). The white fluorescent light, red LED, and white LED provided a statistically identical number of PLBs (Figure 1a). However, red and green LED increased the number of PLBs, up to 71.6% and 81.1%, respectively, over the white fluorescent light (Figure 1a). The average fresh weight was increased to 57.8% and 80.8% by the red and green LED, respectively, over the white fluorescent light (Figure 1b). The average fresh weight was statistically identical among the green, red, and white LED, whereas only the green LED showed as statistically different from white fluorescent light for the average fresh weight (Figure 1b). Blue LED significantly reduced the PLBs regeneration, in number of PLBs and their fresh weight, of *D. okinawense* (Figure 1). Red LED generated the highest number of shoots (0.93) per explant, whereas green and white LED produced 0.67 shoots per explant (Table 1A). Shoot numbers did not show any significant variation among the light sources (Table 1A). All four LEDs (red, white, blue, and green) had a similar shoot formation in terms of percentage (26.7%) (Table 1A), whereas the PLB formation rate was 100% in LEDs (except for white and blue LED) (Table 1A). Our results suggested that green LED would be efficient on *D. okinawense* PLBs regenerations. LEDs might have some role in stimulating (red and green LED) and arresting (blue LED) the PLBs regeneration of *D. okinawense*.

### 2.3. Anti-Auxin, PCIB, for PLB Regeneration

**Figure 2:**
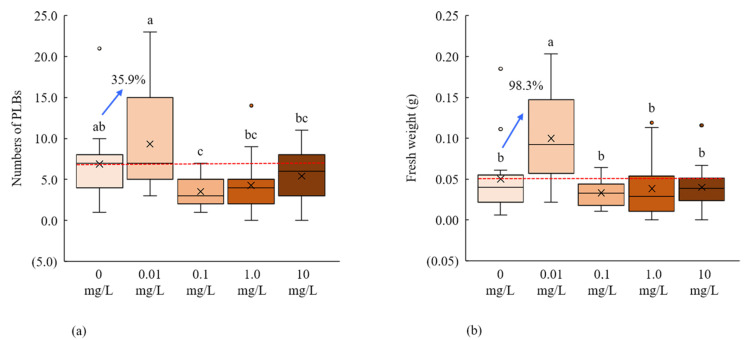
PLBs regeneration of *D. okinawense* in the culture media using different concentrations of PCIB. (**a**) Effect on the number of PLBs regeneration, (**b**) Effect on their fresh weight. Crosses in the box represent the mean number of PLBs and fresh weight (total explant, *n* = 15). Red horizontal lines represent the mean mean value of control (0 mg/L). Blue arrows over the boxplot represent the percentage in increase from red horizontal line. Boxplots that share a letter are statistically identical in Tukey’s multiple comparison test (*p* < 0.05).

We used four different concentrations of PCIB (0.01, 0.1, 1.0, and 10 mg/L) for the PLBs regeneration of *D. okinawense,* whereas culture media without PCIB (0 mg/L) was considered as control. We found that low concentrations of PCIB (0.01 mg/L) significantly increase the number and fresh weight of newly generated PLBs, whereas the subsequent increase of the PCIB concentration (0.1, 1.0, and 10 mg/L) led to the reduction of the PLBs regeneration (Figure 2a). The addition of the 0.01 mg/L PCIB in culture media increased the number of PLBs and fresh weight, at 35.9% and 98.3%, respectively, over control (Figure 2b). The number of PLBs and fresh weight were dramatically decreased with the increase of PCIB concentrations (Figure 2).

Using 0.01 mg/L PCIB in culture media, we determined the maximum number of PLBs (9.3 PLBs per explant), fresh weight (0.10 g), and the number of shoots (2.87 shoots per explant) (Figure 2, Table 1B). We observed 100% PLB formation rate in 0, 0.01, and 0.1 mg/L of the PCIB, respectively, whereas the shoot formation rate was also higher for 0 and 0.01 mg/L over the other concentrations (Table 1B). The result suggested that the addition of a low concentration of PCIB can stimulate the PLBs regenerations of *D. okinawense*.

### 2.4. An Antifungal Agrochemical, HMI for PLB Regeneration of D. okinawense

**Figure 3:**
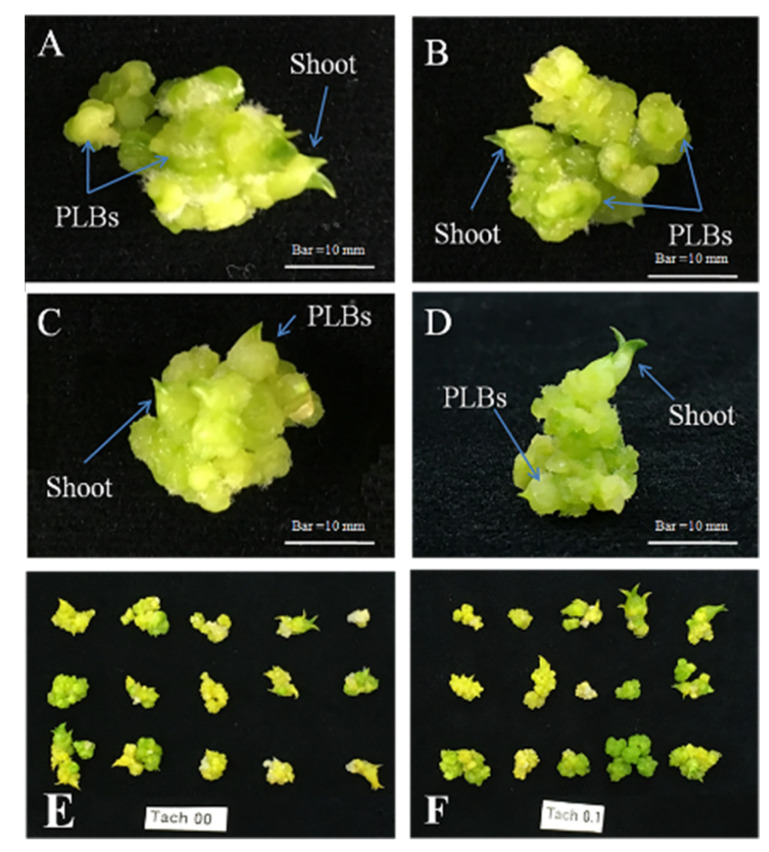
Formation of PLB and shoot of *D. okinawense* at different concentrations of HMI (**A**–**D**) from single PLB). (**A**) 0 mL/L (Control), (**B**) 0.01 mL/L, (**C**) 0.1 mL/L, (**D**) 1.0 mL/L of HMI, (**E**) newly generated PLBs in control from the fifteen mother PLBs, (**F**) newly generated PLBs in 0.1 mL/L from the fifteen mother PLBs.

We used three different concentrations of Tachigaren (or HMI) and considered the culture media without HMI (0 mL/L) as a control for the PLB regeneration of *D. okinawense* (Figure 3).

**Figure 4:**
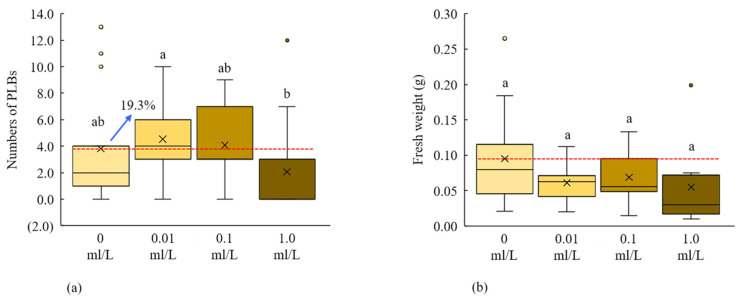
Response of different concentrations of HMI for the PLBs regeneration of *D. okinawense* on (**a**) the number of PLBs and (**b**) fresh weight. Crosses in the box represent the mean number of the newly regenerated PLB from the total explant (*n* = 15). Red horizontal lines represent the mean mean value of control (0 mL/L).The blue arrow over the boxplot represents the percentage from red horizontal line. Boxplots that share a letter are statistically identical in Tukey’s multiple comparison test (*p* *≤* 0.05).

The low concentration of HMI (0.01 mL/L) in culture media significantly increased (19.3%) the number of PLBs over control (Figure 4a). We found 4.5 PLBs per explant from the culture media treated with 0.01 mL/L HMI (Figure 4a). We did not find any significant variation in fresh weight and number of newly generated shoots by the application of different concentrations of HMI (Figure 4b, Table 1C). Application of very low concentrations of HMI can increase the number of PLBs of *D. okinawense.*

### 2.5. Effect of LEDs on PCIB- and HMI-Manipulated Culture Media for the PLB Regeneration

**Figure 5:**
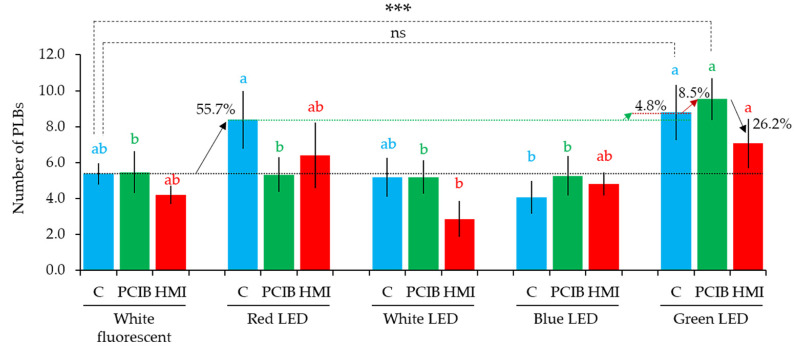
The number of newly generated PLBs of *D. okinawense* under different LEDs using PCIB- and HMI- manipulated culture media. Blue, green, and red bars represent non-manipulated culture media (C), 0.01 mg/L PCIB-manipulated culture media (PCIB), and 0.01 mL/L HMI-manipulated culture media (HMI), respectively (total number of explants, *n* = 15). Values are means ± standard error (s.e.; *n* = 15). Upward arrows represent the percentage of increase from the respective horizontal lines and downward arrow represents the percentage of decrease. Black, green, and dark red dotted horizontal lines represent the mean number of PLBs, respectively, produced by control under white fluorescent light, control under red LED, and control under green LED. The color of the different upward arrows represents the increase from the mean (similar colored dotted horizontal line) level. Letters above the bars that share the same letter are statistically identical and those that do not share the same letter are statistically different at *p ≤* 0.05 using Tukey’s multiple comparison test (significance levels between the same color letters only). Here, ***; *p* *≤* 0.01, ns; not significant by student *t*-test.

A low concentration of PCIB (0.01 mg/L) and HMI (0.01 mL/L) had a significant effect on the PLB regeneration of *D. okinawense* (Section 2.3 and Section 2.4). We have further analyzed the effect of PCIB (0.01 mg/L) and HMI (0.01 mL/L) in culture media under four different LEDs for PLB regeneration of *D. okinawense*. Our PCIB-manipulated culture media did not show any noticeable increase in the number of PLBs when they were cultured under red, white, and blue LED, compared with white fluorescent light while green LED, which significantly increased the number of PLBs (green bars in Figure 5). On the other hand, HMI- manipulated culture media did not show any noticeable increases in the number of PLBs, compared with the white fluorescent light (red bars in Figure 5). PCIB under white LED had no effect while HMI under white LED reduced the number of PLBs, compared to the control under white fluorescent light. PCIB under red LED had no effect whereas HMI under red LED showed a greater number of PLBs compared to control, PCIB, and HMI under white fluorescent light. However, HMI under red LED showed lower number of PLBs compared to control under red LED. In the case of non-manipulated culture media (C), which were free from either PCIB or HMI, the highest number of PLBs produced by being under red LED and green LED (blue bars in Figure 5) showed identical results with our previous result in Figure 1a. Furthermore, under green LED, a non-significant increase was recorded in the number of PLBs between PCIB, HMI, and non-manipulated culture media (C). Culture media manipulated by PCIB and HMI tended to positively affect the regeneration of PLBs under the green LED (Figure 5). However, culture media manipulated by PCIB and HMI under blue LED were similar in the number of PLBs compared with control under white fluorescent light (Figure 5). Therefore, among the five light treatments and the three-culture media, the PCIB-manipulated media resulted in the significantly highest number of PLBs under green LED.

The red LED increased to 55.7% PLBs over the white fluorescent light and further increased 4.8% from the green LED (over the red LED), whereas the white and blue LED reduced the number of PLBs, compared to control (blue bars in Figure 5). The trend of PLBs formation was similar to our LED experiment results (Figure 1). Culture media having PCIB (0.01 mg/L) did not show any significant variation under all the light sources, except in green LED (green bars in Figure 5). Culture media having PCIB under the green LED significantly increased (55.7% + 4.8% + 8.5% = 69%) the number of PLBs over the control under the white fluorescent light (Figure 5). In the culture media having HMI (0.01 mL/L), the red and green LEDs increased while the white and blue LEDs decreased the number of PLBs compared to control (red bars in Figure 5). Our results suggest that both chemicals (PCIB and HMI) have the potentiality to increase the number of PLBs of *D. okinawense* in combination with green LED. Under the green LED, culture media without PCIB produced 8.8 PLBs per explant, whereas the addition of 0.01 mg/L of PCIB in the culture media increased the number of PLBs (8.5%) (Figure 5). Under the green LED, the number of PLBs showed the tendency to increase in the culture media having HMI (0.01 mL/L); however, the number of PLBs was 26.2% lower compared to the 0.01 mg/L of PCIB- manipulated culture media (Figure 5).

**Figure 6:**
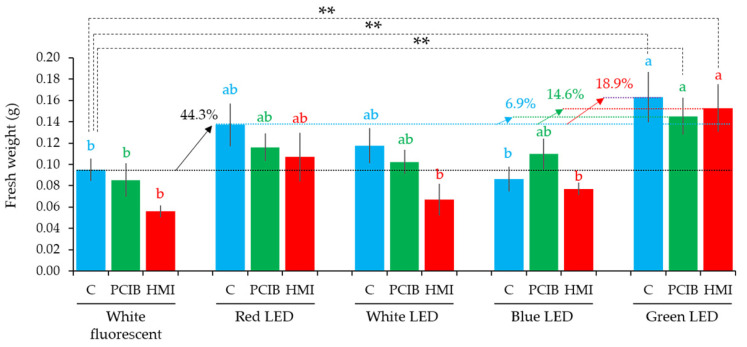
Fresh weight of *D. okinawense* under different LEDs using PCIB- and HMI-manipulated culture media. Blue, green, and red bars represent non-manipulated culture media (C), 0.01 mg/L PCIB-manipulated culture media (PCIB), and 0.01 mL/L HMI-manipulated culture media (HMI), respectively (total number of explants, *n* = 15). Values are means ± standard error (s.e.; *n* = 15). Up-ward arrows represent the percentage of increase from the respective horizontal lines. Black, light blue, green, red, and dark red dotted horizontal lines represent the average fresh weight in control under white fluorescent light, control under red LED, PCIB under green LED, HMI under green LED, and control under green light. Letters above the bars that share the same letter are statistically identical and those that do not are statistically different at *p ≤* 0.05 using Tukey’s multiple comparison test (significance levels between the same color letters). Here, **; *p ≤* 0.05 by student *t*-test.

The green LED significantly increased (44.3% + 18.9% = 63.2%) in fresh weight over the white fluorescent light (Figure 6). PCIB- and HMI-manipulated culture media showed a tendency to increase in fresh weight under the green LEDs, compared to the control under white fluorescent light (Figure 6). In comparison with the control–white fluorescent combination, the PCIB-green–LED combination significantly increased to 51.2% (44.3 + 6.9%) in fresh weight light, whereas it was 58.9% (44.3 + 14.6%) in the HMI-green–LED combination (Figure 6). Culture media with 0.01 mg/L of PCIB and 0.01 mL/L HMI produced the maximum number of shoots (1.53 and 0.47 per explants) under green LED compared to other treatments (Table 2). PLB and shoot formation, as percentages, have been mentioned in Table 2.

## 3. Discussion

MS medium [48] is usually used in micropropagation for diverse orchids including the *Dendrobium* genus [49,50]. Sucrose is widely accepted for diverse plant species as the major carbohydrate source to supply energy to cells in plant tissue culture media [51]. The concentration of sucrose is also species specific for micropropagation, while 20 g/L sucrose is used in orchid tissue culture [34,41,52,53,54]. We confirmed that the use of 20 g/L of sucrose can be efficient for the PLB regeneration of *D. okinawense*, just as with any other *Dendrobium* sp. (Supplementary Table S1) [8,9]. Sucrose is needed in plant embryos to increase cell division and encourage cell expansion [55,56]. The ideal concentration of sucrose is important for micropropagation because sucrose deficiency/oversupply can be detrimental to plant cell growth in vitro. We also found lower PLBs regenerations of *D. okinawense* by very high or very low concentrations of sucrose (Supplementary Table S1). Lower sucrose hampers the carbon supplies to the in vitro explant, whereas high sugar concentration can lead to osmotic stress conditions, or an excessive carbohydrate accumulation that ultimately hampers the photosynthesis process [56].

Light is a signaling component that controls plant morphology, physiology, growth, and development both in vivo and in vitro [57,58,59,60,61,62]. Excessive light can lead to cell damage during in vitro plant propagation [63,64]. Light-emitting diodes (LED) are more effective than the traditional fluorescent light, but are also species specific [65]. In this study, we identified the specific monochromatic LED for the in vitro PLB regeneration of *D. okinawense*. Generally, plants respond well to red and blue LED, whereas green LED is hardly ever considered for the plants’ biomass growth; moreover, red and blue wavelengths are strongly absorbed by chlorophyll a and b [26]. *D. okinawense* showed the most efficient PLB regeneration under green LED and subsequently under red LED, whereas PLB regeneration of *D. okinawense* negatively responded under blue LED (Figure 1, Figure 5 and Figure 6, Table 1). Optimistic and undesirable PLB regeneration of the *D. okinawense* under green and blue LED, respectively, indicated the plant specificity for micropropagation to dissimilar monochromatic light. However, we did not check the monochromatic light combination for the PLB regeneration of *D. okinawense,* which may have the possibility for the further improvement of the PLB regeneration. Previous studies also reported the efficiency of PLB regeneration using the combination of different monochromatic lights [4,5,41]. The effectiveness of red and blue light is well known, whereas very few studies have reported the efficacy of the green light in vitro. Green LEDs have discrete effects on plant biology [66] and the specific mechanisms of green light have not been identified yet. The leaves of the plants appear green, which does not mean that plant reflects all green light; only 10–50% of green light is reflected by plant chloroplasts [26]. The rest of the green light has been absorbed by chlorophyll and carotenoid pigments, and the absorbed green light is also used for photosynthesis. The existence of the plant growth response to green light has also previously been found by some researchers [67,68], but this response is not easily attributed to the currently known suite of light receptors [66,69]. Green wavelength can penetrate the plant canopy; it can drive and regulate different plant physiological processes, stimulate photosynthesis, contribute to carbon gain, and ultimately lead to more biomass production [26,70]. Green wavelengths cannot be ignored in LED-based in vitro plant propagation or crop cultivation in horticulture.

PCIB plays an important role in plant growth and development by auxin perception and signal transduction due to their auxin-induced physiological responses inhibition characters [13,14]. The in vitro callus formation, PLBs regeneration, and root-shoot development can be promoted by the application of exogenous auxin, and auxin in conjunction with cytokinins as well [18,71]. We found that the addition of a very low concentration of PCIB (0.01 mg/L) in MS medium in vitro greatly enhanced the regeneration of PLBs of *D. okinawense*, as well as their fresh weight and number of shoots (Figure 2). On the other hand, the regeneration of *D. okinawense* was decreased by the application of more than 0.01 mg/L of PCIB, which was even lower than the control (Figure 2). This study revealed that the application of a low concentration of PCIB can play a role in in vitro PLBs regeneration of *D. okinawense*. The culture media manipulated by 0.01 mg/L of PCIB–green LED responded better for the PLB regeneration compared with the PCIB–fluorescent light (Figure 6). It suggests to us that PCIB is more effective for PLB regeneration, in combination with other factors, such as green LED (our findings) and ABA [72]. Fungal contamination is a serious problem in micropropagation and different toxic chemicals, such as sodium hypochlorite (NaOCl) or mercury chloride (HgCl_2_), are used to disinfect plant materials. We used Tachigaren (HMI) in culture media for the PLB regeneration of *D. okinawense*. Tachigaren is an antifungal agent that can control fungal growth; additionally, it can regulate plant growth and development by increasing auxin activity [16]. We found that a very low concentration of HMI (0.001 mL/L) in culture media increases the number of PLBs over control. The PLBs regeneration was chronologically decreased with the increases of HMI concentration. In addition, at high HMI concentration (1 mL/L), the PLBs numbers were even lower than control (Figure 3). The 0.001 mL/L HMI-manipulated culture media produced 4.2 to 4.5 PLB per explant, whereas the number of PLB increased to 7.1 per explant under green LED (Figure 3 and Figure 4). Tachigaren was also found to be more effective in aggregation with other factors, such as a green LED, for the in vitro PLB regeneration in *D. okinawense*.

In general, plants are more responsive under red and blue light for in vitro propagation. As is the case with universal knowledge for in vitro plant propagation, we also found red LEDs to be a promoting factor in in vitro PLB regeneration of *D. okinawense*. Interestingly, we found green LED instead of blue LED as a promoting factor for PLB growth in *D. okinawense*. We cannot disregard the lesser influencing green LED for the plant micropropagation, specifically in PLB regeneration of orchids. However, the effect of monochromatic light can be species specific. PLB regeneration of *D. okinawense* can be accelerated by a very low concentration of PCIB or HMI in culture media specifically under monochromatic green or red light. We found a significant level of increase in PLB proliferation by the 0.01 mg/L of PCIB and green LED combination over the control. Our results suggested that green LED in conjunction with a low concentration of anti-auxin (PCIB) or antifungal agent (HMI) can be 60–70% more effective for the PLBs regenerations of *D. okinawense* compared to the usual application of red or blue LED. Our current findings will help to conserve the endangered epiphytic orchid, *D. okinawense*, by rapid PLBs regeneration. However, we need more studies using diverse combinations of growth promotors, growth inhibitors, elicitors, and LEDs (specifically green and red LED) for the most efficient PLBs regeneration of *D. okinawense*.

## 4. Materials and Methods

### 4.1. Modification of MS Media, pH Adjustment, and Solidification

Ammonium nitrate (412.5 mg/L) and potassium nitrate (950.0 mg/L) were used to modify the MS medium, and this modification was followed in the culture media preparation for subcultures and experiments [45,73]. The pH of modified MS media was between 5.5–5.8. Then, we added 1 mM 2-(N-morpholino) ethanesulfonic acid sodium salt (MES-Na) buffer. Thereafter, we adjusted the pH at 5.7 either 0.1 N NaOH or 0.5 N HCl before autoclaving. A total of 20 g/L sucrose (Sigma-Aldrich^®^, Tokyo, Japan) was used in the culture media except for the sucrose-specific treatments. In total, 2.2 g/L Phytagel^TM^ (Sigma-Aldrich^®^) was used to solidify the culture medium. The culture medium was autoclaved at 121 °C for 15 min at 117.1 KPa.

### 4.2. Preparation of Explants

PLBs of *D. okinawense* were generated from the rhizome following the protocol developed by Shimasaki and Uemoto [73] and multiplied in the modified Murashige and Skoog (MS) medium. In two-month intervals, PLBs were sub-cultured and transferred into the new culture medium, having similar medium composition. For the experiment, PLBs were excised into single PLBs and every single PLB was used as an explant. Visually similar-sized PLBs were used for the experiment.

### 4.3. Volume of Culture Media, Number of Explants in Each Culture Vessel, and Photoperiod

A total of 50 mL of culture media in a 250 mL culture vessel (UM culture bottle: AsOne, Osaka, Japan) was used for the primary PLB generations and their subculture. In each of the independent experiments, 30 mL of culture media was used in a 250 mL culture vessel, 5 explants were inoculated in the culture vessel, and 3 culture vessels (repetitions) were used for each treatment. The culture chamber was adjusted to a 16-hour photoperiod; the temperature was set at 25 ± 1 °C temperature throughout the experimental period. Except for the specific light treatments, PLBs were cultured under a white fluorescent tube (FL20SS, Toshiba, Tokyo, Japan), with an average of 54 μmolm^−2^s^−1^ photosynthetic photon flux density.

### 4.4. Evaluation of the Sucrose Level for PLB Regeneration

PLBs proliferation efficiency was evaluated using 0, 5, 10, 20, 30, and 40 g/L sucrose in the culture media, respectively. The modification of MS media, pH adjustment, solidification, volume of culture media, number of explants in each culture vessel, and photoperiod conditions are described in Section 4.1 and Section 4.3.

### 4.5. Manipulation of the Environment and Culture Media

#### 4.5.1. Manipulation of the Environment by Light-Emitting Diodes

PLBs were regenerated under four different monochromatic lights (LEDs) with white fluorescent tubes as control. Four LEDs were white (wavelength: 420–750 nm, product code: P18W-E1701-W, Jefcom, Osaka, Japan), red (wavelength: 580–670 nm, product code: P18W-E1701-R, Jefcom), blue (wavelength: 420–550 nm, product code: P18W-E1701-B, Jefcom), and green (wavelength: 460–610 nm, product code: P18W-E1701-G, Jefcom). The average photosynthetic photon flux density for monochromatic LEDs was 54 μmolm^−2^s^−1^.

#### 4.5.2. Manipulation of the Culture Media by Anti-Auxin and Antifungal Agent

Two independent experiments, one for an anti-auxin and another for an antifungal agent, were conducted. Culture media were manipulated by the different concentrations of an anti-auxin PCIB (0, 0.01, 0.1, 1, and 10 mg/L) (Sigma-Aldrich^®^,) and an antifungal agent HMI (0, 0.01, 0.1, and 1.0 mL/L) (Sigma-Aldrich^®^) (Tachigaren^TM^, first launched by Sankyo Co., Ltd.; and currently as Mitsui Chemicals Agro, Inc., Tokyo, Japan). The commercial PCIB was in water-soluble powder form and the HMI was in liquid form. PCIB was dissolved in sterile water due to the difficulties in measuring its small amount. The respected volume of dissolved PCIB and commercial HMI was directly added into the culture media before autoclaving. PLBs were then cultured under a white fluorescent tube with an average of 54 μmolm^−2^s^−1^ photosynthetic photon flux density.

#### 4.5.3. PCIB and HMI Manipulated Culture Media under LEDs

For the PLB multiplication, the best concentration of PCIB and HMI were identified from two independent experiments described in Section 4.5.2. Culture media were again manipulated independently by PCIB and HMI. Only the best concentration (from experiments described in Section 4.5.2) of PCIB and HMI for the number of PLBs was used and then cultured under four LEDs (white, red, blue, and green) with white fluorescent light as control. Wavelengths and photosynthetic photon flux density for the LEDs are explained in Section 4.5.1.

### 4.6. Data Collection and Analysis

Experiments were organized in a completely randomized design with five biological and three technical replications. Three culture vessels, having five explants in each vessel, were used for each of the single experiments. Each explant in each culture vessel was considered a biological replication, and the culture vessel was considered a technical replication. All experiments were considered as completely randomized designs. PLBs were cultured for forty-two days in the culture chamber for each independent experiment. The number of PLBs and number of shoots were counted, and their fresh weights were measured at forty-two days of culture in each independent experiment. Only the mature and well-developed PLBs were counted (Figure 3A–D), whereas the newly formed and tiny PLBs were not counted in each independent experiment. The average numbers and percentages of PLBs and shoots were calculated as follows:

Average number = Total number of newly generated PLB ÷ total number of explants

PLB (%) = (Number of explants that generated new PLBs ÷ total number of explants) × 100

Shoot (%) = (Number of explants that generated shoots ÷ total number of explants) × 100

Data of fifteen explants were used for the statistical analysis considering each as a replication. The data were statistically analyzed by one-way ANOVA using agricolae package in R (https://www.R-project.org/web/packages/agricolae/, accessed on 1 March 2022) and using Tukey’s multiple comparison test at 95% confidence interval.

## Figures and Tables

**Table 1:** PLBs proliferation percentage (%), shoot formation in percentage (%), and number of shoots regenerated from different culture conditions and media manipulation.

(A) Different LEDs			
LEDs	PLBs (%)	Shoot (%)	Shoot numbers
WF	100.0	20.0	0.40 ± 0.27 a
R LED	100.0	26.7	0.93 ± 0.50 a
W LED	93.0	26.7	0.67 ± 0.33 a
B LED	80.0	26.7	0.40 ± 0.19 a
G LED	100.0	26.7	0.67 ± 0.41 a
(B) Different PCIB concentrations under white fluorescent light			
PCIB (mg/L)	PLBs (%)	Shoot (%)	Shoot numbers
0	100.0	40.0	2.07 ± 0.09 a
0.01	100.0	40.0	2.87 ± 1.32 a
0.1	100.0	6.7	0.40 ± 0.40 b
1	86.7	13.3	0.67 ± 0.46 b
10	93.3	6.7	0.27 ± 0.27 b
(C) Different HMI concentrations under white fluorescent light			
HMI (mL/L)	PLBs (%)	Shoot (%)	Shoot numbers
0	86.7	40.0	0.47 ± 0.17 a
0.001	86.7	26.7	0.60 ± 0.32 a
0.01	93.9	53.3	0.67 ± 0.21 a
0.1	60.0	13.3	0.20 ± 0.14 a

Here, data are represented as the mean numbers ± SE from the total explant cultured (*n* = 15). WF; White fluorescent light, R; red, W; White, B; Blue, and G; Green. Treatments that share a letter are statistically identical in Tukey’s multiple comparison test (*p* < 0.05).

**Table 2:** PLBs proliferation percentage (%), shoot formation percentage (%), and number of shoots regenerated under different LEDs.

(A) Culture media having PCIB (0.01 mg/L)			
LEDs	PLBs (%)	Shoot (%)	Shoot numbers
WF	100.0	20.0	0.33 ± 0.21 b
R LED	93.3	26.7	0.47 ± 0.24 b
W LED	86.7	40.0	0.67 ± 0.25 b
B LED	86.7	33.3	0.60 ± 0.27 b
G LED	100.0	33.3	1.53 ± 0.88 a
(B) Culture media having HMI (0.001 mL/L)			
LEDs	PLBs (%)	Shoot (%)	Shoot numbers
WF	100.0	NSF	NSF
R LED	73.3	13.3	0.13 ± 0.09 a
W LED	46.7	13.3	0.13 ± 0.09 a
B LED	100.0	6.7	0.20 ± 0.12 a
G LED	93.3	20.0	0.47 ± 0.26 a

Here, data are represented as the mean numbers ± SE and as a percentage from the total explant cultured (*n* = 15), NSF; no shoot formation. WF; White fluorescent light, R; red, W; White, B; Blue, and G; Green. Treatments that share a letter are statistically identical using Tukey’s multiple comparisons test (*p* < 0.05).

## Data Availability

Not applicable.

## Supplementary Materials

The following supporting information can be downloaded at: https://www.mdpi.com/article/10.3390/plants11081082/s1, Table S1: Effectiveness of different concentrations of sucrose for PLB regeneration of *D. okinawense*.
