# Supplementary material for: Low Concentration of Anti-Auxin and Anti-Fungal Agent Accelerates the PLB Regeneration of Dendrobium okinawense under Green LED
Source: Plants (Basel). 2022 Apr 15;11(8):1082. doi: 10.3390/plants11081082 (PMC9028245; doi:10.3390/plants11081082)
Supplement: Supplementary file 1 [file plants-11-01082-s001.zip › plants-1642357-supplementary.pdf]

Table S1. Efficiency of sucrose concentrations for PLB organogenesis of *D. okinawense.*

Sucrose (g/L)	Mean Number of PLBs	Fresh Weight (g)	Mean Number of Shoots	PLBs Formation Rate
0	3.20 c ± 0.26	0.0267 d ± 0.00174	0.47 b ± 0.16	100%
5	5.12 ab ± 0.35	0.076 bc ± 0.00577	1.12 ab ± 0.22	100%
10	5.60 a ± 0.45	0.099 a ± 0.00723	1.83 a ± 0.29	100%
20	5.17 a ± 0.39	0.080 ab ± 0.00654	0.90 b ± 0.19	100%
30	5.33 a ± 0.49	0.060 bc ± 0.00548	1.30 ab ± 0.25	100%
40	3.33 bc ± 0.23	0.055 c ± 0.00300	1.13 ab ± 0.23	100%

Here; values are means ± standard error (s.e.; n = 15); letters in the column that share a letter are statistically identical at $p \leq 0.05$ by Tukey's multiple comparisons test at 95% confidence interval.
